# The Use of an Activity Monitoring System for the Early Detection of Health Disorders in Young Bulls

**DOI:** 10.3390/ani9110924

**Published:** 2019-11-05

**Authors:** Mohammed Anouar Belaid, Maria Rodriguez-Prado, Eric Chevaux, Sergio Calsamiglia

**Affiliations:** 1 Animal Nutrition and Welfare Service, Department of Animal and Food Sciences, Universitat Autònoma de Barcelona, 08193 Bellaterra, Spain; anouarbelaid@gmail.com (M.A.B.); maria.rodriguez.prado@uab.cat (M.R.-P.); 2 Lallemand SAS, R&D Animal Nutrition, 31702 Blagnac, France; echevaux@lallemand.com

**Keywords:** beef cattle, activity monitoring, behavior, diseases

## Abstract

**Simple Summary:**

In large intensive beef production systems, the identification of sick animals is difficult. We hypothesized that sick bulls would change daily activities when sick. Thus, the use of activity monitoring devices might allow for the early identification of sick bulls. The device used measured steps counts, lying time, lying bouts, and frequency and time at the feed bunk. Sick bulls started to behave differently from healthy bulls at least 10 days before the appearance of clinical signs. The prediction model identified bulls at risk of becoming sick 9 days before the visual diagnostic based on the time attending to the feed bunk, the time lying, and the frequency of lying bouts. The validation indicated that the prediction resulted in 50% false positives and 7% false negatives. Activity monitoring systems may be useful tools to identify bulls at risk of becoming sick.

**Abstract:**

Bulls (n = 770, average age = 127 days, SD = 53 days of age) were fitted with an activity monitoring device for three months to study if behavior could be used for early detection of diseases. The device measured the number of steps, lying time, lying bouts, and frequency and time of attendance at the feed bunk. All healthy bulls (n = 699) throughout the trial were used to describe the normal behavior. A match-pair test was used to assign healthy bulls for the comparison *vs*. sick bulls. The model was developed with 70% of the data, and the remaining 30% was used for the validation. Healthy bulls did 2422 ± 128 steps/day, had 28 ± 1 lying bouts/day, spent 889 ± 12 min/day lying, and attended the feed bunk 8 ± 0.2 times/d for a total of 95 ± 8 min/day. From the total of bulls enrolled in the study, 71 (9.2%) were diagnosed sick. Their activities changed at least 10 days before the clinical signs of disease. Bulls at risk of becoming sick were predicted 9 days before clinical signs with a sensitivity and specificity of 79% and 81%, respectively. The validation of the model resulted in a sensitivity, specificity, and accuracy of 92%, 42%, and 82 %, respectively, and a 50% false positive and 12.5% false negative rates. Results suggest that activity-monitoring systems may be useful in the early identification of sick bulls. However, the high false positive rate may require further refinement.

## 1. Introduction

Mortality and morbidity at arrival are common in beef production systems [1]. Owners and managers of cattle feedlots are constantly seeking management practices to reduce their incidence to improve profitability. Antibiotics are frequently used in this period to prevent diseases [2]. However, the use of antibiotics in animal production has the potential to increase antimicrobial resistance in humans [3,4]. Therefore, there is an urgent need to develop strategies to reduce the use of antibiotics without compromising health, production, or animal welfare [5].

The early identification of sick animals may allow for an early and targeted therapy and, eventually, decrease antibiotic use [6]. However, in large intensive feedlot operations, the early identification of bulls with health problems by farm personnel is difficult if it is based only on observations.

Recently, a variety of automatic activity monitoring devices have been developed and can assist farm personnel in the early detection of diseases [7,8]. Feeding behavior has been identified as a major behavioral change when young cattle get sick, and because changes occur 4 to 6 days before the diagnostic by farm personnel, it can be used for the early detection of diseases [9,10,11]. Most of this research focused only on feeding behavior, but other activities were not recorded. We hypothesized that the use of feeding behavior, together with other activities, would improve the ability of monitoring systems to identify animals at risk of becoming sick earlier. Therefore, the objective of this study was to determine if the feeding behavior, together with steps counts, lying time, and lying bouts, could be useful in the early identification of newly received bulls at risk of becoming sick.

## 2. Materials and Methods

### 2.1. Animals, Housing, and Management

Crossbred bulls (n = 770; average age = 127 days; standard deviation = 53 days of age) originating from auction markets in northern Spain (National Cattle Market, Torrelavega) were supplied to the receiving facility and monitored during the first three months after their arrival. The experiment was conducted from July 2016 to March 2018. There were four groups of bulls that arrived at the farm in July (n = 33), March (n = 212), June (n = 252), and October (n = 273). All groups were managed in an all-out all-in program between groups. On the day of arrival, bulls were vaccinated against Pasteurella and pneumonia (Neo-bacterina^®^, Syva, Ciudad Real, Spain) and were treated with an internal and external anti-parasitic treatment (Paramectin^®^, Syva, Ciudad Real, Spain). Seven days after the first vaccine, all bulls were revaccinated. Bulls were housed in a dirt floor open-air facility with an average space of 7 m^2^/bull, and had continuous access to water and dry food. The concentrate (14% crude protein, 4% fat) was offered in a 6 m long feed bunk, straw in a separate 15 m long feed bunk, and water was provided through an automatic waterer. Bulls were monitored in the lots for 12 ± 2 weeks.

### 2.2. Data Collection 

At arrival, bulls were immediately weighed and fitted in the front right leg with an activity monitoring device (Fedometer [FEDO] system; ENGS, Rosh Pina, Israel). The device consists of an accelerometer that measured daily steps counts (n/day), lying bouts (n/day), and lying time (min/day). An electromagnetic field-generating antenna was located along the feed bunk to detect bulls which front leg was less than 30 ± 2 cm from the feed bunk to allow the calculation of the number of visits (n/day) and its duration (min/day) at the feed bunk. Data collected was transmitted wirelessly every 6 min to a computer with a system-specific software (Eco-herd-software; ENGS, Israel). The system has been described in detail and validated for feedlot cattle [12]. Bulls ID, birth date, body weight, and group entry date were recorded for each animal. Bulls were observed daily by experienced farm personnel for clinical signs following a checklist of criteria including appetite, fecal consistency, respiratory efforts, hydration status, and general attitude. All criteria were scored as normal (0), mild (1), or severe (2). A bull was diagnosed with a respiratory disease when observed with a fast or difficult breathing, coughing, nasal mucus, or ocular discharge; with digestive problems when they had signs of diarrhea (feces with a loose to watery consistency and strong odor), presence of loose fecal matter on the top of the tail or legs, and/or dehydration; and with locomotion problems if they had difficulties in walking. When one severe or two mild criteria were observed, rectal temperature was measured, and, if > 39.7 °C, the disease was confirmed. Diagnostic and treatments were conducted under the attending veterinarian supervision within 12 h of the identification of the sick animal. Antibiotics were used to treat respiratory problems, and the rest were treated with a combination of antibiotics and anti-inflammatory drugs for at least three consecutive days. The date, the time of treatment, and the treated diseases were recorded for each morbidity event. The system was checked periodically for proper functioning. 

### 2.3. Statistical Analyses

All statistical analyses were performed with SAS (v.9.4; SAS Institute Inc., Cary, NC, USA). For the normal behavior, only bulls that had no treatment during all the study were used. A descriptive analysis was conducted for the entire period, and univariate analysis (ANOVA) was performed to identify differences by age for each behavioral index. Significance was declared at *p* < 0.05 and trends reported at 0.05 ≤ *p* < 0.10.

A matched pair design was used to assign bulls to the healthy group for comparisons. For each sick bull, we identified three healthy bulls that were on the same date, the same group, and similar age (±4 d) and weight at entry (±17 kg). For sick bulls that were treated multiple times, only the first treatment event was considered. Day 0 was defined as the day of sickness diagnosis, and the database contained records from day −10 to day +10. Once the database was generated, a multivariate linear mixed model was built to describe differences for each behavioral index between sick and healthy bulls. The model included bulls health status (healthy, sick), season (summer, autumn, winter, and spring), age at entry, days around the treatment event (from −10 to +10), and the interaction between days and health status as fixed effects. The animal was considered a random effect. When an interaction was significant, the SLICE option of SAS was used to evaluate these differences.

For the development and validation of the model, 70% of the original dataset was used to develop the prediction model, and the remaining 30% was used to measure the performance of the model. To build the prediction model, multivariate logistic regression was performed on the days before the first treatment event to identify variables that were associated with health status. This model included the fixed effects of age at entry, season, and all behavior indexes. Age at entry was included in all prediction models. All predictors with a *p* < 0.20 were initially included in the model. The backward stepwise procedure was used to exclude variables from the regression model until all predictors remaining were significant. For each model, the sensitivity (Se) and the specificity (Sp) were calculated for each possible cutoff point, as described by Dohoo et al. [13]. The cutoff point that yielded the highest combination of Se and Sp was selected, and the model score was defined. Diagnostic test characteristics included the false positive rate (FPR), the false negative rate (FNR), and accuracy [13]. The Se was defined as the proportion of sick bulls that were correctly diagnosed. The Sp was defined as the proportion of healthy bulls that were correctly diagnosed. The FPR was defined as the proportion of calves that were diagnosed incorrectly sick. The FNR was defined as the proportion of calves that were diagnosed incorrectly healthy. Once all prediction models were generated on the days before the first treatment event, the one with the highest area under the curve (AUC) was chosen to evaluate its performance in the validation dataset (30% remaining from the original database). The model was applied on the same day as the validation dataset to obtain the FPR and FNR.

## 3. Results

### 3.1. Normal Behavior 

**Figure 1:**
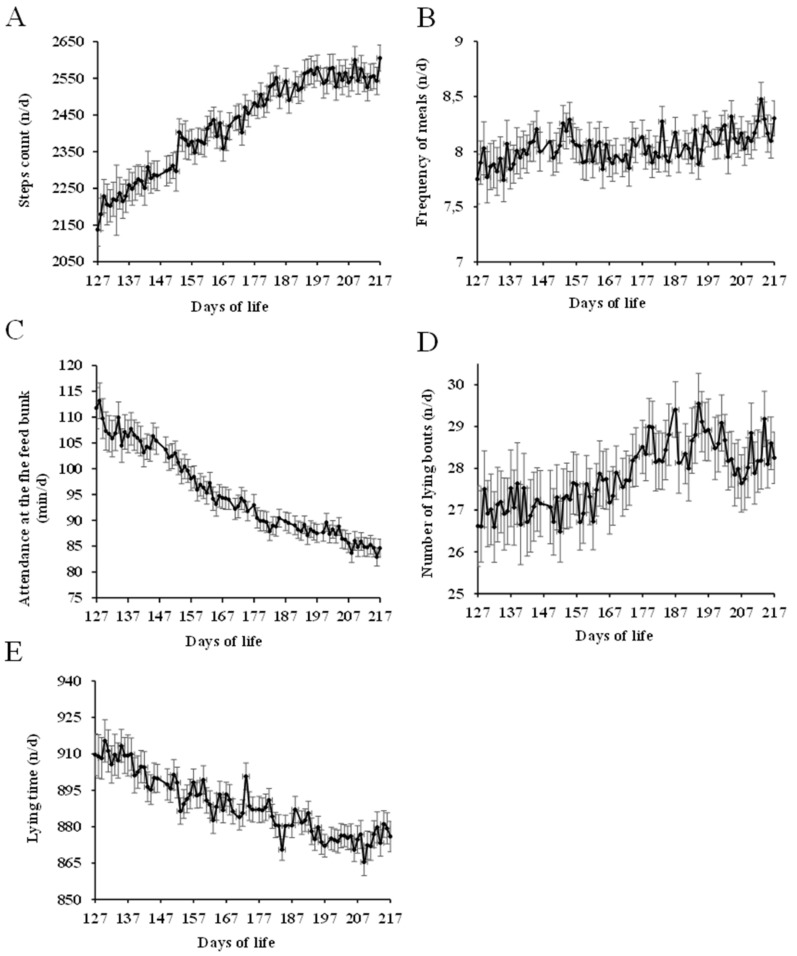
Daily steps counts n/day (**A**), frequency of meals n/day (**B**), attendance at the feed bunk min/day (**C**), number of lying bouts n/day (**D**) and lying time min/day (**E**) in healthy bulls from 127 to 217 days of life.

Only bulls that had no treatment during all the study were used to describe the normal behavior (n = 699; Figure 1). Bulls age ranged from 127 to 217 days, did an average of 2422 ± 128 steps/day, had 28 ± 1 lying bouts/day, spent 889 ± 12 min/day lying, and attended the feed bunk 8 ± 0.2 times/day for a total of 95 ± 8.2 min/day. Age was significant in all behavior indexes (*p* < 0.05) except in the frequency of meals where no differences were observed (*p* > 0.10). Bulls daily average in step counts and lying bouts (Figure 1A,D) increased (*p* < 0.05), and attendance to the feed bunk and lying time (Figure 1C,E) decreased, with age (*p* < 0.05). 

### 3.2. Differences in Behavior Between Healthy vs. Sick Bulls

**Figure 2:**
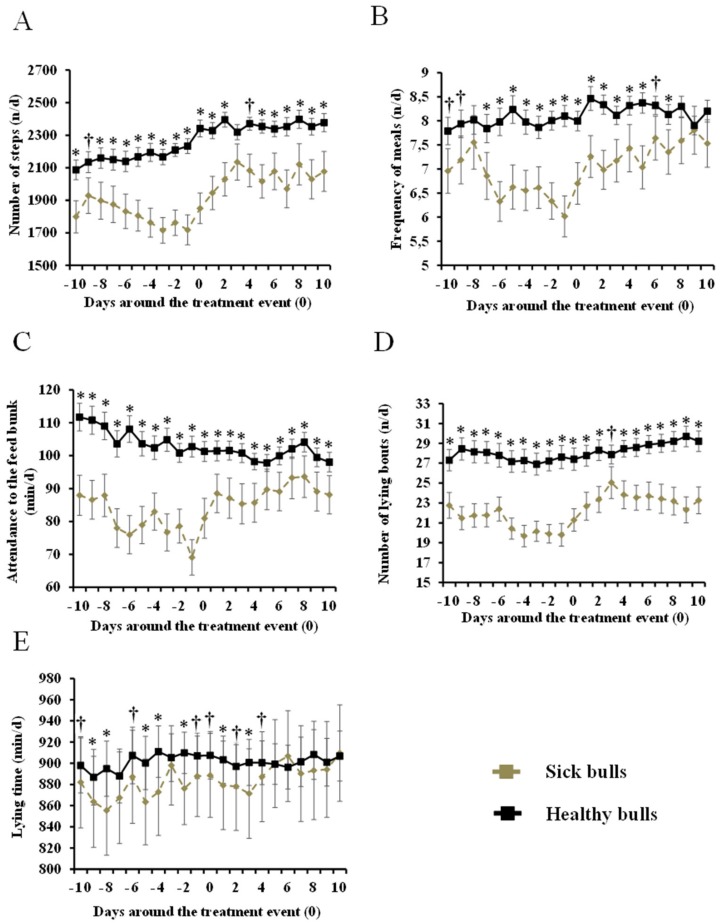
Daily steps counts n/day (**A**), frequency of meals at the feed bunk n/day (**B**), attendance at the feed bunk min/day (**C**), number of lying bouts n/day (**D**), and time lying min/day (**E**) for of sick bulls (n = 33) *vs*. matched paired healthy bulls (n = 99) from 10 days before to 10 days after the first treatment event. Means within day differ (*; *p* < 0.05) or tended to differ (†; 0.05 < *p* < 0.10).

Of the total of 770 bulls enrolled in the study, 71 had at least one episode of sickness, which represents an overall incidence of 9.2%. The mortality rate during this experiment was 1.2% of enrolled bulls. Figure 2 shows the comparison between sick and healthy bulls (n = 71 *vs.* 213, respectively) in all behavior measurements from 10 days before to 10 days after the first treatment event. All behavior indexes were affected by bull’s health status. 

Differences between healthy and sick bulls started to be evident from at least 10 day before the first treatment event took place. Sick bulls did in average 15% fewer steps (days: −10, −8, −7, −6, −5, −4, −3, −2, −1, 0, +1, +2, +5, +6, +7, +8, +9, and +10; *p* < 0.05; −9 and 4; *p* < 0.10), 22% less lying bouts (from days −10 to +10 except for day +3; *p* < 0.05; day +3; *p* < 0.10), and spent less time lying (days: −9, −8, −5, −4, −2, +1, and +3; *p* < 0.05; −10, −6, −1, 0, +2, and +4; *p* < 0.10) compared with healthy bulls. Bulls attended the feed bunk 15% fewer times (days: −7, −6, −5, −4, −3, −2, −1, 0, +1, +2, +3, +4, +5, and +7; *p* < 0.05; −10, −9 and +6; *p* < 0.10), and spent 18% less time at the feed bunk (days: from −10 to +10; *p* < 0.05) than healthy bulls. Table 1 provides the probability level for the effect of health status, days of sickness, season, age, and the interaction between health status and days of sickness on all behavioral indexes.

### 3.3. Predictive Models and Validation

The match-pair design is a useful tool to assign healthy bulls to control for environmental and site-specific factors affecting behavior [14]. All prediction models for the days before the first treatment event had an AUC > 0.75. Table 2 shows the outcomes of the diagnostic test characteristic resulting from all the prediction models of the training database from the days before the diagnostic of sick bulls. The best model with the highest AUC value (0.88) was found for day −9. This model at the cut-off point chosen from the highest combination of Se (79%) and Sp (81%) had 81%, 47%, and 6% accuracy, FPR, and FNR, respectively. The predictive model of day −9 included attendance at the feed bunk, lying bouts, and lying time as behavior indexes remaining significant after the backward stepwise procedure. The predictive logistic regression model obtained on day −9 was used in the validation dataset to measure its performance and resulted in a sensitivity, specificity, and accuracy of 92%, 42%, and 82 %, respectively. The FPR and FNR were 50% and 12.5%, respectively. Table 3 summarizes the result of the analysis of maximum likelihood estimates for the parameters of the model for day −9.

## 4. Discussion

### 4.1. Normal Behavior 

One of the objectives of the present study was to describe the bull’s normal daily behavior. A good understanding of bull’s normal behavior is necessary to establish references from which we can distinguish bulls at risk of becoming sick. There are few reports where all activities have been measured and reported at the same time. Most previous studies have monitored only some activities separately and will serve as reference for the discussion of the normal behavior of healthy bulls. In general, the results reported herein agree with previous reports. For example, bulls in our study did, on average, 2422 ± 128 steps/day, and agree with results reported by Devant et al. [15] for healthy bulls before castration (2544 ± 120 steps/day). Pillen et al. [16] observed a lower value of daily steps (1472 steps/day). However, the value was the average of bulls and steers, and considering that steers have 56% lower activity compared with bulls [15], the estimated value for bulls was close to 2000 steps/day. The lying bouts have not been as well studied as the rest of behavioral indexes. We found only three studies reporting results that varied widely. In our study, bulls did, on average, 27.8 ± 0.76 lying bouts/day. Rouha-Muelleder et al. [17] reported a wide variation of lying bouts in bulls depending on the type of flooring and ranged from 9.1 to 28.3 lying bouts/day. In contrast, Pillen et al. [16] and Ball et al. [18] reported that bulls did, on average, from 11.4 to 15 bouts/day. Regarding total lying time, bulls in our study spent 889 ± 13 min/day lying and agrees with other reports where healthy bulls spent almost 60% of their daily time lying [19,20,21]. For feeding behavior, most previous reports also support our findings. Bulls in our study attended the feed bunk 8 ± 0.15 times/day and for a total of 95 ± 8 min/d. Similar studies also found that bulls attended the feed bunk 7.8 to 10.5 times [22] and 97 min/day [19]. While we monitored the attendance at only the concentrate feed bunk, other reports monitored access to the concentrate and straw feed bunk together. However, because the diets offered in all studies cited previously were composed of almost 90% concentrate, the comparison was considered valid.

### 4.2. Differences in Behavior Between Healthy vs. Sick Bulls

To our knowledge, the present research is the first study that compared five different daily activities at the same time from days −10 to +10 relative to the diagnostic of the disease between sick and healthy bulls. Most studies focused predominantly on feeding behavior, but not on other activities, for the early prediction of diseases in bulls [9,10,22].

Activity and behavior have been long considered indicators of pain and illness [23]. Sick bulls react to the illness reducing their activity, and they could be easily distinguished from healthy bulls. Hanzlicek et al. [24] reported that sick heifers reduced steps counts after inoculation with *Mannheimia haemolytica*. Similarly, White et al. [25] inoculated calves with *Mycoplasma bovis* and observed that steps counts were negatively associated with the appearance of clinical signs of the diseases. In our study, the difference between healthy and sick bulls in steps counts was already evident 10 days before the first treatment event took place. Marchesini et al. [11] observed that bulls suffering from bovine respiratory syndrome (BRD) had lower activity and rumination 3 to 6 days before the onset of visible signs of the diseases. Similarly, Pillen et al. [16] in newly received feedlot cattle reported that calves had a lower activity 6 days before BRD diagnosis, and this reduction was more pronounced the day before the diagnosis. Therefore, activity may be useful as an early predictor of health disorders.

For most of the activities monitored, the largest difference compared with healthy bulls occurred the day when farm personnel identified the animal as potentially sick (Figure 2). This provides evidence of the reliability of the protocol followed by farm personnel in identifying sick bulls. Sick bulls herein had lower lying bouts from day −10 until day +10 relative to the first treatment. Swartz et al. [14] observed that lying bouts in young calves suffering from BRD began to decrease 2 days before having any sign of sickness, and continued until 3 days after treatment. Pillen et al. [16] also found that lying bouts of sick bulls decreased from 14.5 to 11.4 compared with healthy bulls. Sutherland et al. [26] observed that the number of lying bouts in sick calves with neonatal diarrhea tended to decrease 5 days before the clinical diagnostic of the disease. These previous results are in agreement with our results, demonstrating that the lying bouts are affected before and after the first treatment event. Sick bulls spent less time lying than healthy bulls, and this reduction was more pronounced before the appearance of clinical signs of sickness than after. There is no general agreement on the effect of sickness on lying time in bulls. Sutherland et al. [26] reported that calves before having neonatal diarrheas spent more time lying 2 days before clinical signs but less time lying the day of the diseases appearance. In contrast, Theurer et al. [27] observed an increase in lying time on calves after inoculation with *Mannheimia haemolytica* compared with before infection. In spite of the lack of agreement in the literature on the effect of diseases on lying time, in our study, lying bouts and lying time remained in the prediction model after a backward stepwise selection of variables, as discussed later. Our results also demonstrate that feeding behavior was affected before and after the disease diagnostic, where the frequency of attendance and the total time spent at the feed bunk was reduced in sick bulls. Several studies agree with our findings. For example, Sowel et al. [28] found that healthy steers spent 30% more time at the feed bunk compared with morbid steers. Sowel et al. [22] also reported in a follow-up study that sick steers attended the feed bunk less frequently than healthy steers. Buhman et al. [29] observed that the frequency and the time of attendance at the feed bunk were lower in sick calves compared with healthy calves. Quimby et al. [9] found that the feeding behavior was able to identify sick animals 4.1 days earlier than the diagnosis by the farm personnel. Wolfger et al. [10] reported that steers with BRD had a lower daily frequency of meals (12 *vs*. 9.7) and shorter time (9.7 *vs*. 7.6 min) per meal 7 days before feedlot staff noticed any clinical sign.

Overall, sick bulls showed changes in daily activities already at 10 days before the diagnostic of the disease. We monitored bulls behavior from 10 days before the sickness based on previous studies that observed changes in behavior 2 to 6 days before the observation of clinical signs [9,11,14,16,26]. However, results suggest that these changes may start occurring even earlier, and future studies should monitor activities for periods longer than 10 days.

### 4.3. Predictive Models and Validation

To our knowledge, our study is the first to develop a model to predict diseases in bulls with a formal validation using an independent dataset. Because we considered it important to select the earliest date for an early treatment, the analysis was conducted on individual days. According to our results, the prediction model developed using the training database on day −9 had a Se and an Sp of 79% and 81%, respectively. The Se and the Sp were similar to the results found in previous studies predicting different diseases. For example, Marchesini et al. [11] reported that Se and Sp of the prediction model from days 1 to 3 before the clinical diagnostic for the BRD and lameness in beef cattle were 81% and 95%, respectively. Quimby et al. [9] used feeding behavior to predict BRD in newly received calves and found a Se of 78% and an Sp of 79% of the model at day 6 before the visual detection of the diseases. Wolfger et al. [10] also used feeding behavior to predict BRD and reported a Se of 82% and a Sp of 78% on day 5 before the observation of clinical signs. The prediction model included time of attendance to the feed bunk, lying bouts, and lying time as behavioral predictors. Surprisingly, measures of activity (step counts), in spite of the clear differences between healthy and sick bulls (Figure 2), did not provide additional information to the model. 

The application of the prediction model at day −9 in the independent dataset (validation data) resulted in an acceptable overall accuracy (82%). However, the low specificity (42%) resulted in a high FPR (50%). The FPR represents the percentage of animals that would be treated without any need. However, the high FPR should be interpreted in the context of current preventive practices. In many farms, preventive treatments are conducted in all animals upon arrival. In the current experiment, the incidence of diseases was around 10%. A 50% FPR implies that twice as many animals (20%) would be treated. However, when preventive treatments are commonly provided to all animals [1], the strategy proposed will reduce by 80% the unnecessary preventive treatment and, therefore, will reduce the use of antibiotics. Furthermore, early detection may also improve the effectiveness of treatments and the consequences of the disease, reducing the use of antibiotics and the negative economic impact of the disease, and improving the wellbeing of bulls. On the other hand, the FNR (12.5%), that represents the percentage of animals that would not be treated when they actually need treatment, while acceptable, implies that periodic surveillance of animals to catch those that are not identified by the prediction model is still required.

## 5. Conclusions

Activity monitoring devices may be useful tools for the early identification of bulls at risk of becoming sick. The most reliable prediction model was able to identify these bulls 9 days before the visual detection of clinical signs by farm personnel. However, the high FPR found could affect the reliability of this prediction, which deserves further refinement.

## Figures and Tables

**Table 1:** The probability level for the effect of health status, days before sickness, season, and the interaction between health status and days of sickness on the daily steps counts (n/day), frequency of meals at the feed bunk (n/day), attendance at the feed bunk (min/day), number of lying bouts (n/day), and lying time (min/day) for sick bulls (n = 71) *vs*. matched paired healthy control bulls (n = 213).

Predictive Variables	Steps Counts	Frequency of Meals	Attendance to the Feed Bunk	Lying Bouts	Lying Time
	*p*-Value	*p*-Value	*p*-Value	*p*-Value	*p*-Value
Season	<0.05	<0.10	0.12	>0.05	<0.05
Age	<0.05	<0.05	<0.05	>0.05	>0.05
Days	<0.05	<0.05	<0.05	<0.05	<0.05
Health status	<0.05	<0.05	<0.05	<0.05	<0.05
Days × health status	>0.05	>0.05	<0.05	<0.10	>0.05

**Table 2:** Outcomes of the diagnostic test characteristic resulting from the prediction models of the training database from days before the treatment event of sick bulls.

Days ^1^	AUC ^2^	Accuracy, %	Se ^3^, %	Sp ^4^, %	FPR ^5^, %	FNR ^6^, %
−10	0.86	80	84	78	42	7
−9	0.88	81	79	81	47	6
−8	0.79	75	72	76	57	9
−7	0.81	71	74	71	61	9
−6	0.83	70	80	67	62	7
−5	0.80	66	69	66	66	11
−4	0.76	70	69	71	63	10
−3	0.86	79	82	78	50	6
−2	0.87	76	83	75	55	6
−1	0.84	75	76	75	56	8

^1^ Days before the sick bull diagnostic. ^2^ Area under the curve. ^3^ Sensitivity. ^4^ Specificity. ^5^ False positive rate. ^6^ False negative rate.

**Table 3:** The analysis of maximum likelihood estimates for the parameters of the prediction model for d −9.

Parameter	Estimate	Standard Error	*p*-Value
Intercept	8.512	3.1704	< 0.05
Age at entry, days	0.017	0.0090	> 0.05
Attendance to the feed bunk, min/day	−0.036	0.0112	< 0.05
Lying bouts, n/day	−0.127	0.0429	< 0.05
Lying time, min/day	−0.006	0.0030	< 0.05
